# Measurement of Mean Cardiac Dose for Various Breast Irradiation Techniques and Corresponding Risk of Major Cardiovascular Event

**DOI:** 10.3389/fonc.2014.00284

**Published:** 2014-10-22

**Authors:** Tomas Rodrigo Merino Lara, Emmanuelle Fleury, Shahram Mashouf, Joelle Helou, Claire McCann, Mark Ruschin, Anthony Kim, Nadiya Makhani, Ananth Ravi, Jean-Philippe Pignol

**Affiliations:** ^1^Radiotherapy Unit, School of Medicine, Pontificia Universidad Católica de Chile, Santiago, Chile; ^2^Department of Radiation Oncology, Sunnybrook Odette Cancer Centre, Toronto, ON, Canada; ^3^Department of Radiation Oncology, University of Toronto, Toronto, ON, Canada; ^4^Department of Radiation Oncology, Erasmus MC, Rotterdam, Netherlands

**Keywords:** breast neoplasms, radiotherapy, heart diseases, brachytherapy, radiation dosage

## Abstract

After breast conserving surgery, early stage breast cancer patients are currently treated with a wide range of radiation techniques including whole breast irradiation (WBI), accelerated partial breast irradiation (APBI) using high-dose rate (HDR) brachytherapy, or 3D-conformal radiotherapy (3D-CRT). This study compares the mean heart’s doses for a left breast irradiated with different breast techniques. An anthropomorphic Rando phantom was modified with gelatin-based breast of different sizes and tumors located medially or laterally. The breasts were treated with WBI, 3D-CRT, or HDR APBI. The heart’s mean doses were measured with Gafchromic films and controlled with optically stimulated luminescent dosimeters. Following the model reported by Darby (1), major cardiac were estimated assuming a linear risk increase with the mean dose to the heart of 7.4% per gray. WBI lead to the highest mean heart dose (2.99 Gy) compared to 3D-CRT APBI (0.51 Gy), multicatheter (1.58 Gy), and balloon HDR (2.17 Gy) for a medially located tumor. This translated into long-term coronary event increases of 22, 3.8, 11.7, and 16% respectively. The sensitivity analysis showed that the tumor location had almost no effect on the mean heart dose for 3D-CRT APBI and a minimal impact for HDR APBI. In case of WBI large breast size and set-up errors lead to sharp increases of the mean heart dose. Its value reached 10.79 Gy for women with large breast and a set-up error of 1.5 cm. Such a high value could increase the risk of having long-term coronary events by 80%. Comparison among different irradiation techniques demonstrates that 3D-CRT APBI appears to be the safest one with less probability of having cardiovascular events in the future. A sensitivity analysis showed that WBI is the most challenging technique for patients with large breasts or when significant set-up errors are anticipated. In those cases, additional heart shielding techniques are required.

## Introduction

In developed countries, breast cancer is the most common type of cancer in women (2, 3). With implementation of mammographic screening, the majority of the cases are diagnosed at an early stage. The standard treatment for early stage breast cancer includes removing the tumor and sampling the axillary lymph nodes using limited surgery (4). This is followed by whole breast radiotherapy and possibly regional radiation if nodes are positive. Currently, there is a general trend toward treatment de-escalation. Radiation oncology studies demonstrate that the duration of whole breast irradiation (WBI) can be shortened from 6 to 3 weeks (5–7), and other showing that for selected cases the amount of irradiated breast tissue could be limited to a small portion surrounding the surgical cavity (8, 9). This leads to a technique called accelerated partial breast irradiation (APBI). It combines a reduction of the irradiated breast volume and delivery of higher dose per fraction. Multiple APBI techniques have been proposed including external beam 3D-conformal radiotherapy (3D-CRT), high-dose rate (HDR) interstitial brachytherapy using multicatheter or balloon, and permanent breast seeds implants (10, 11). As a result, patients with early stage breast cancers are treated with a variety of radiation techniques that appears comparable in terms of effectiveness (12, 13).

Along with the changes in radiation oncology practice mentioned above, the increased early detection of breast cancer due to screening programs has also resulted into improvement of the breast cancer treatment outcomes, with specific survival rates of 98.6% at 5 years (14). With improved survival, the reduction of treatment induced morbidity and mortality has gained importance as they may eliminate the need for adjuvant radiotherapy. Several studies with long-term follow-up have shown that standard external beam radiotherapy can increase the risk of ischemic heart disease and a recent large case control study suggests that a dose–response relationship between the mean dose to the heart and the long-term risk of major cardiovascular events including mortality (1, 15–18). It is unknown if all the radiation techniques used in early stage breast cancer have similar cardiac risks since there are no long-term prospective data comparing them on this specific outcome. There is a limited number of studies reporting or comparing the heart dose (19–25) for one or two techniques but there has not been thorough comparison of the mean dose to the heart for all breast techniques, for various breast sizes and/or seroma locations. In most of cases, commercial treatment planning systems (TPS) are used for estimation of the heart’s dose. Since this dose is calculated outside the field where photon scattering dominates, some concern about the accuracy of those calculations exists (26, 27).

Given the inaccuracies in calculating out of field dose with the current clinical TPS, the purpose of this study was to measure and compare the mean heart dose for different breast irradiation techniques delivered to the left breast of an anthropomorphic phantom. In addition, the robustness of our findings was tested using a sensitivity analysis looking at the added influence of breast size, seroma location, and organ motion.

## Materials and Methods

### Preparation of phantoms

**Figure 1:**
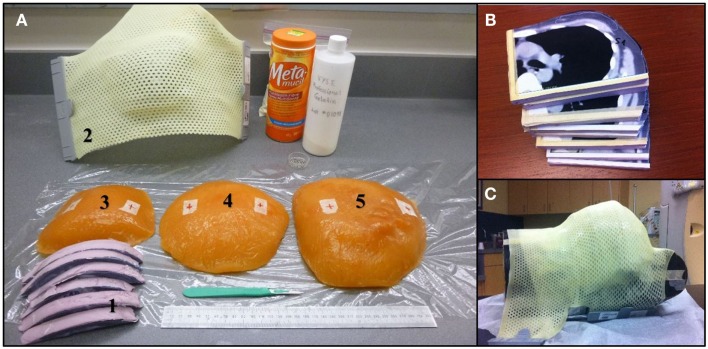
**(A)** Materials used during the breast phantom manufacture. (1) Styrofoam slices cut to fit CT contours. (2) Thermoplastic 3D breast contour obtained from the Styrofoam mold. (3–5) Small, medium, and large size of gelatin-based breast phantoms. **(B)** Styrofoam slices cut to the patient profile using CT images. **(C)** Thermoplastic mold over the Rando phantom modified with the large breast to ensure good contact.

An anthropomorphic Rando phantom (The Phantom Laboratory, Salem, NY, USA) was modified using molded pieces of a tissue equivalent gel for mimicking various breast sizes. In order to prepare the necessary phantom, three CT scans of patients with left sided breast cancer having typically small (300 cc), medium (800 cc), and large breast (1200 cc) volumes were selected from our institution’s dosimetry database. Each CT slice was spaced by 1 cm and was printed on a scale 1:1 and used to create a realistic 3D breast shape assembling several styrofoam sheets of 1 cm thickness. The printout of the patient contour was pasted on individual styrofoam sheets and cut following the chest wall and breast contours (Figure 1). A negative breast mold was then made using a thermoplastic sheet. This negative mold was then filled with a tissue equivalent powdered ballistics gelatin (Vyse, Schiller Park, IL, USA) dissolved in water. The breast phantom was refrigerated overnight. The resulting gelatin phantom has an average CT number of 24 Hounsfield units (HU), which is similar to fibroglandular breast tissue. The phantom was kept at 5°C to limit melting and water evaporation. It was tightly fixed on the Rando phantom chest wall for planning and treatment.

### Treatment simulation and volume definition

Treatment simulation for small, medium, and large breasts was done following standard institution protocol (28). The Rando phantom with various breast volumes placed on the torso was positioned on a breast board. Five radio-opaque beads and/or pen marks were placed on the skin in the lateral, medial, inferior, and superior aspects of the chest to ensure treatment reproducibility. CT slices of 5 mm spacing and 5 mm thickness were acquired with a Philips CT scanner (Philips Healthcare, Andover, MA, USA) and transferred to either the Pinnacle 3 (RaySearch Americas Inc., Garden City, NY) or the Oncentra Brachytherapy planning systems (Nucletron Elekta, Stockholm, Sweden).

For WBI the clinical target volume (CTV) was defined as whole breast gel phantom limited by the Rando chest wall and a 5 mm layer below the phantom surface. For APBI, the CTV were defined either on the medial or the lateral quadrants of the breast. To ensure comparison of similar target volumes, CTVs of 60 cc were delineated. For brachytherapy, the planning target volume (PTV) included a 1.5 cm expansion from the CTV but limited to the Rando chest wall and 5 mm below the breast surface, while for 3D-CRT APBI the PTV included an expansion of 2.5 cm, similarly to the NSABP-B39 protocol (29).

### Treatment protocols

#### External beam radiotherapy

Whole breast irradiation was planned following standard breast IMRT protocol (30) using a prescription dose of 50 Gy in 25 fractions. For the small and the medium-sized breast phantoms, the beam energy was 6 MV, while a mix of 6 and 18 MV beams was used for the large breast volume. In this protocol, a multileaf collimator (MLC) is used to shape several field-in-field beams to compensate for missing tissue and to improve the dose distribution homogeneity. Plans were normalized to a prescription point set at mid-separation, 2/3 of the distance between skin and a base of the tangential fields. Heart shielding involved ensuring the anterior heart volume was away from the posterior beam edge. Standard treatment set-up procedures were followed including verification of each field using portal imaging.

**Figure 2:**
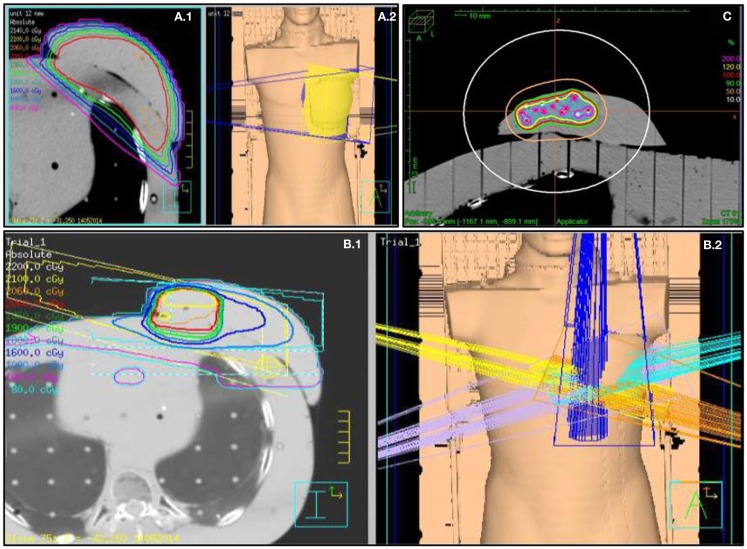
**Fields arrangement and various breast treatments dosimetry**. **(A.1)** Medium size WBI treatment dosimetry and **(A.2)** tangential fields 3D representation. **(B.1)** 3D-CRT APBI dosimetry and **(B.2)** five fields no coplanar 3D representation. **(C)** Multicatheter HDR APBI dosimetry.

For 3D-CRT APBI, three to five non-coplanar beams were aimed (Figure 2) at the PTV (29, 31) and a dose of 38.5 Gy in 10 BID fractions was prescribed. The distribution was normalized on the PTV centroid.

Treatments were delivered using a 6/18 MV Elekta Synergy linac equipped with a multileave collimator (Elekta Inc., Crawley, UK). Treatment was delivered after verification of the correctness of the set-up using portal imaging.

#### Brachytherapy

**Figure 3:**
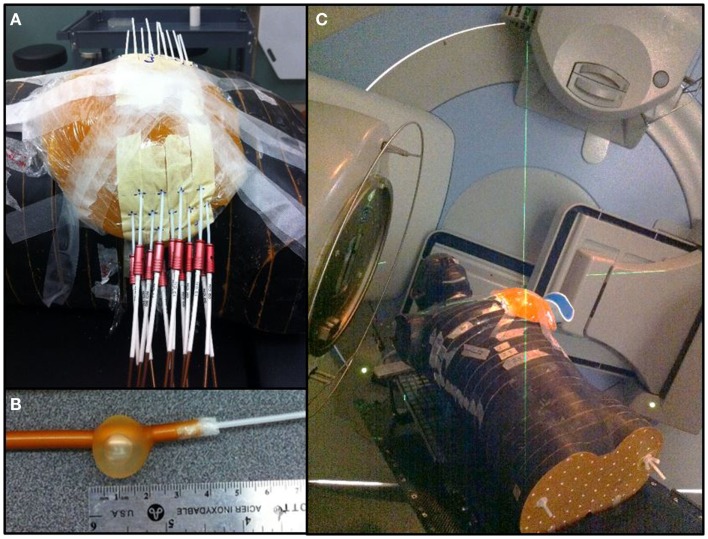
**Breast treatments**. **(A)** Multicatheter HDR APBI. **(B)** Foley catheter used for balloon HDR APBI. **(C)** 3D-CRT APBI.

Using a free-hand technique, 13 catheters were inserted (Figure 3) in a triangular pattern and evenly spaced by 1.5 cm in the horizontal plane and 1 cm in the vertical plane (32). The implanted Rando phantom was CT simulated and the images were transferred to the planning system for target segmentation and dose optimization. A dose of 34 Gy in 10 fractions was prescribed on the minimal peripheral dose (MPD) and dwell times were optimized using the IPSA optimization module (33) to ensure that at least 90% of the target volume (D_90_) will receive at least 90% of the prescribed dose, and that the volume receiving more that 200% of the prescribed dose (V_200_) would be <20 cc. HDR brachytherapy was delivered using a ^192^Ir HDR remote afterloader (Flexitron, Elekta, Stockholm, Sweden). To replicate a balloon catheter HDR treatment, a 3 cm diameter surgical cavity was made in the breast gel phantom and a Foley catheter was positioned inside before being filled with saline. A single catheter was inserted into the Foley catheter and used to deliver a dose of 34 Gy in 10 fractions at the point located 1 cm from the balloon surface.

### Placement of dosimeters

**Figure 4:**
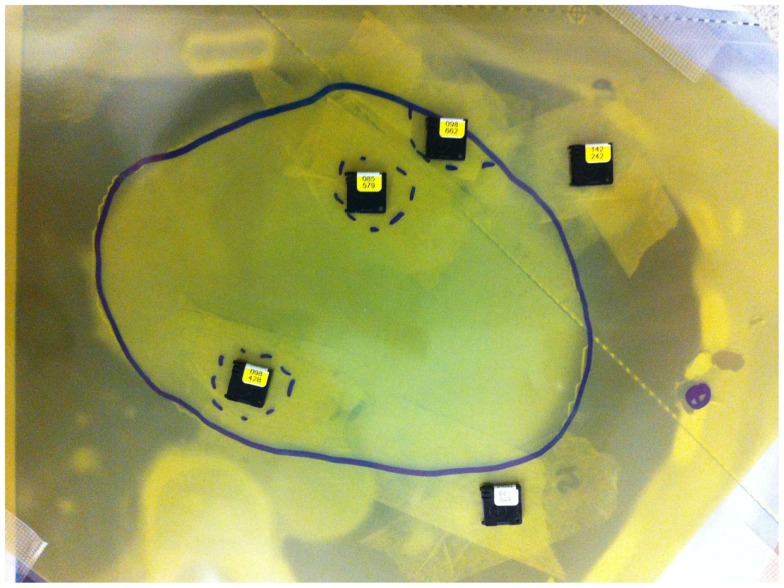
**Optically stimulated luminescent dosimeters and Gafchromic film placement between Rando slices with a 5 mm bolus**.

Two types of dosimeters were used for dose measurements, optically stimulated luminescent dosimeter (OSLD) high-accuracy Nanodot dosimeters (Landauer Inc., Glenwood, IL, USA) and Gafchromic EBT3 films (Ashland Inc., Covington, KY, USA). OSLD dosimeters were placed in areas corresponding to a left descending artery, and the center of left and right ventricles. The detectors were placed inside a bolus material between the three consecutive Rando phantom slices where the heart was identified (Figure 4).

Three Gafchromic films were used to evaluate the heart’s dose distribution in 3D. They were positioned at different location evenly spaced by 1.5 cm. In total, 24 films were irradiated at the three films positions (Apex, medium heart, and base of the heart). For the each technique, films were placed between slices inside the anthropomorphic phantom and fixed in clearly established positions for every experiment. To indicate the exact position of the contour of the heart, the heart contour was drawn on the film with a permanent marker.

In accordance with recommendations of AAPM TG55, the Gafchromic films were kept in a dry and dark area at room temperature for at least 24 h before reading. The heart contours identified on the films were segmented and the optical density was found using the Epson Expression 10000XL scanner (EPSON Deutschland GmbH, Meerbusch, Germany). Optical densities were converted into dose using a calibration curve. All measured dose were expressed as a percentage of the prescribed dose. The doses measured with the 2D film were assumed to represent the average dose absorbed in the adjacent heart’s volume and cumulative DVH were built.

### Sensitivity analysis

Except the used radiation techniques, other changing factors such as the breast size, shape, and location of the seroma, and distance between a heart and a field’s border or a HDR source could also impact on the mean heart dose (34). A meaningful evaluation of the mean heart dose should also account for potential patient set-up error, also called inter-fraction error, for anatomical factors such as heart volume variations between the systolic and diastolic phases or due to patient’s phenotype. To evaluate the impact of those variations for various radiation techniques, the Gafchromic films were reanalyzed shifting the heart position by 1.5 cm. This value is the average of the distance between the field border and the tip of the heart measured on portal imaging for the worse case scenario group in Goody’s study (35). In this report, 11% of the 128 patients had the heart protruding in the irradiation field from 10 to 20 mm.

### Estimation of major cardiac events

Following the model reported by Darby (1), major cardiac were estimated assuming a linear risk increase with the mean dose to the heart of 7.4% per gray (95% confidence interval, 2.9–14.5; *p* < 0.001). Those major cardiac events include myocardial infarction, coronary revascularization, and death from ischemic heart disease, but angina episodes are not included.

## Results

### Quality assurance

The OSLD dose measurements performed inside breast of various sizes were in very good agreement with those calculated with Pinnacle TPS. The dose measured using three to five detectors placed inside the breast was 95% (SD = 2.5%) of the calculated one for the small breast, and 101% (SD = 0.8%) of the calculated one for the medium size breast.

**Figure 5:**
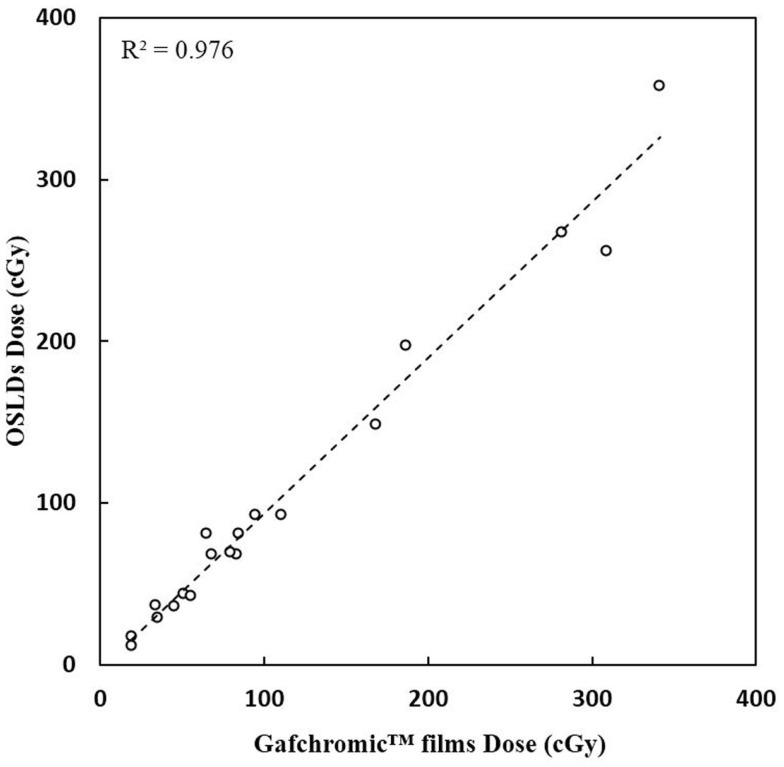
**Correlation between OSLDs and Gafchromic films measurements**.

A very good agreement between OSLD measurements and the Gafchromic film measurements were received (Figure 5). A correlation coefficient *R*^2^ of 0.98 (*p* < 0.001) is calculated.

### Mean dose to the heart

**Figure 6:**
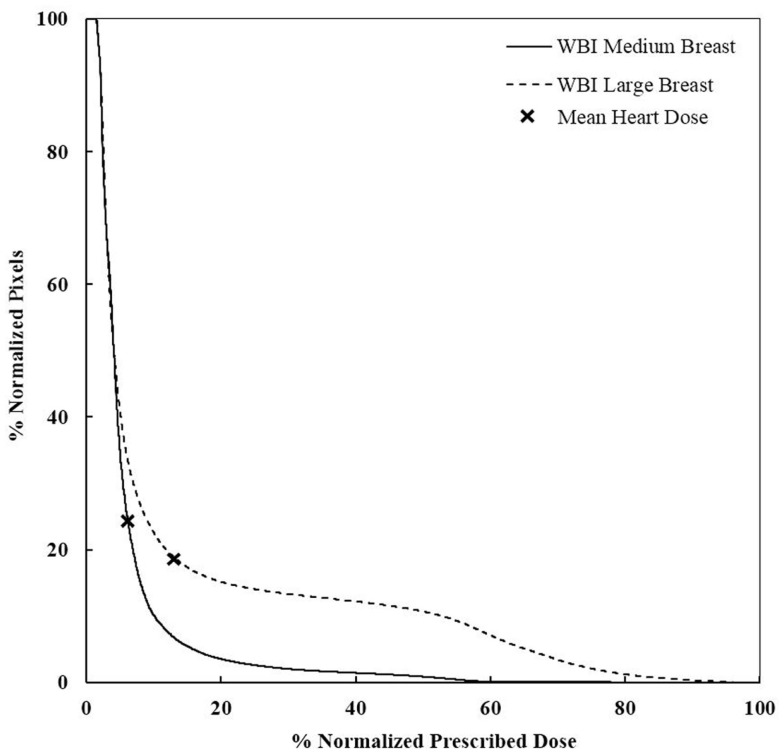
**DVHs for WBI of medium and large breasts**. More heart is receiving a higher dose for large breasts. WBI, whole breast irradiation.

**Figure 7:**
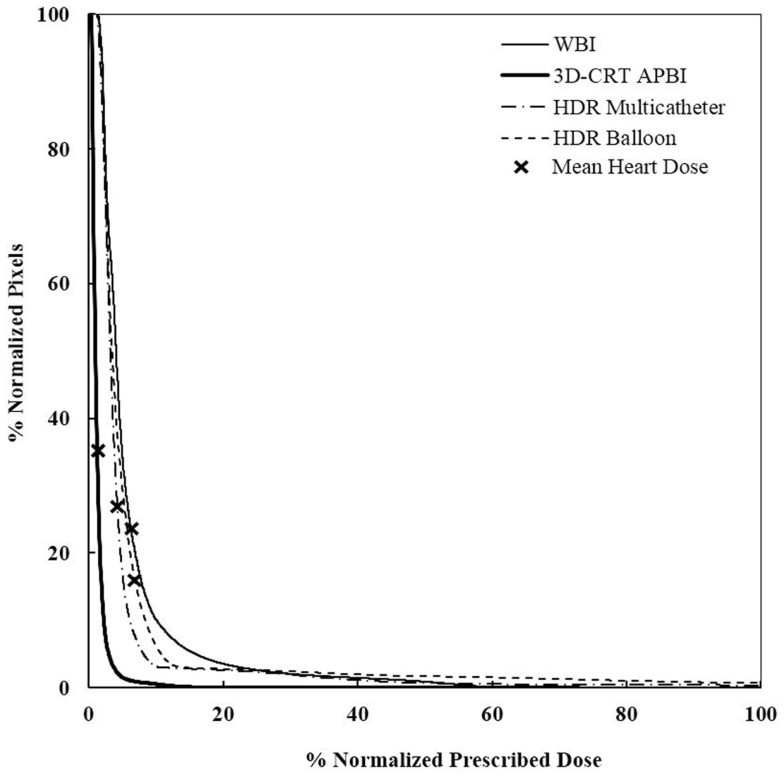
**Cumulative DVHs for various adjuvant breast irradiation techniques for a medially located tumor and a medium breast**. The 3D-CRT APBI appears to be the safest by far. WBI, whole breast irradiation; 3D-CRT APBI, beam 3D-conformal radio therapy accelerated partial breast irradiation; HDR, high-dose rate.

**Figure 8:**
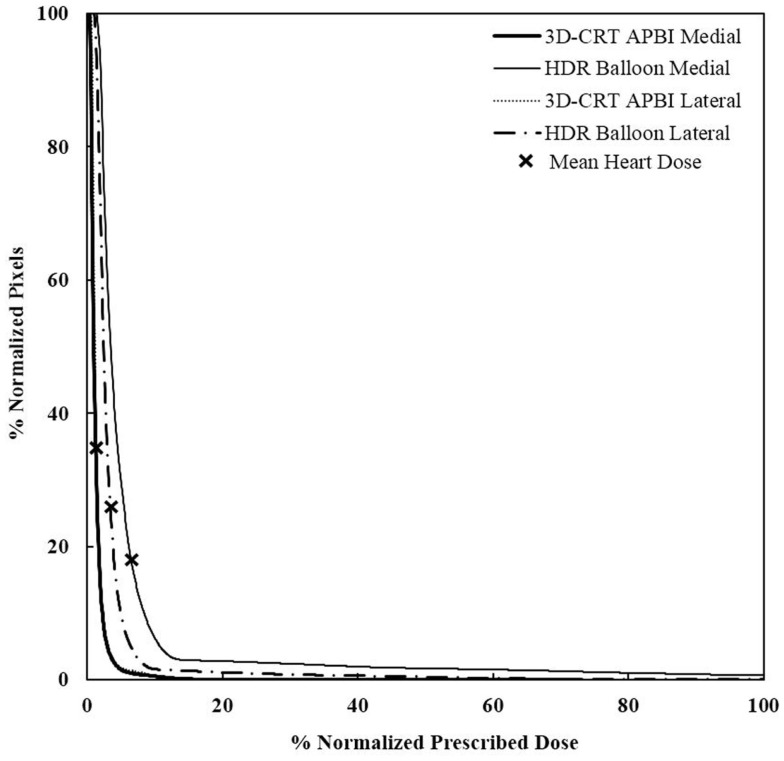
**Cumulative DVHs for various tumor locations and APBI techniques**. There is no impact of location for 3D-CRT as opposed to HDR techniques. 3D-CRT APBI, beam 3D-conformal radio therapy accelerated partial breast irradiation; HDR, high-dose rate.

**Figure 9:**
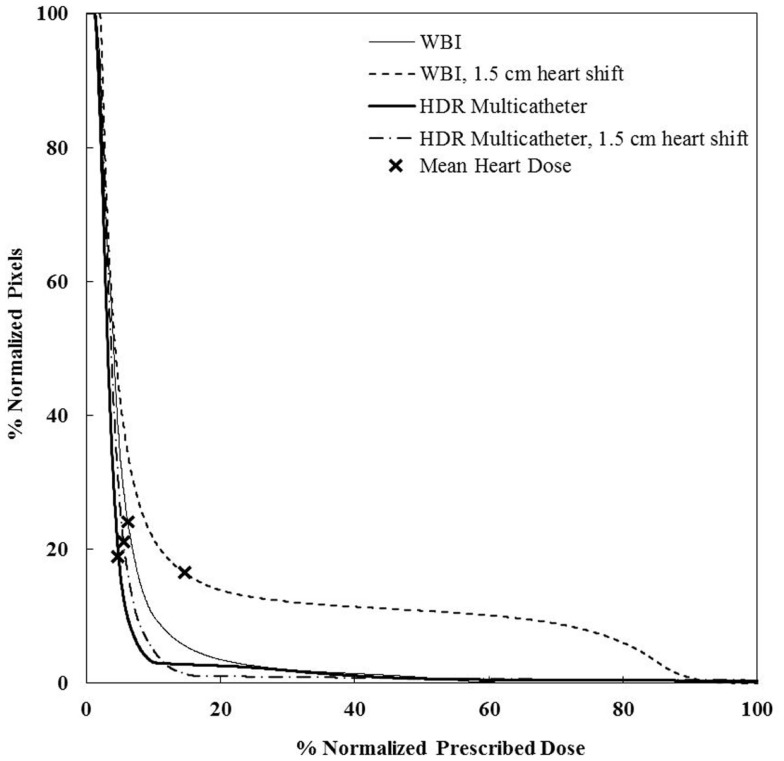
**Cumulative DVHs for the sensitivity analysis on set-up error and motion effect for a medium sze breast and a medially located seroma**. There is little impact of those factors for HDR, but a dramatic effect for WBI. WBI, whole breast irradiation; HDR, high-dose rate.

The measured mean heart’s doses received with different irradiation technique for medium size breast are shown in Table 1. WBI yielded the highest mean heart dose, 2.9 Gy, leading to an estimated increased risk of major coronary events of 22%, while the lowest mean heart dose was measured for the 3D-CRT APBI, 0.5 Gy, leading to a negligible 4% increased risk of cardiac events. The summarized cumulative DVHs for different radiation techniques and different anatomical structures are shown on Figures 6–9.

**Table 1:** **Mean heart dose measured with Gafchromic films for the medium (800 cc) and large breast (1200 cc) phantom using different radiation techniques**.

Technique	Mean dose (Gy)	Relative to prescribed dose (%)	Increased risk of coronary events in % (95% CI)^b^
**WBI**			
Medium (800 cc)	2.99	5.99	22.0 (8.7–43.4)
Large (1200 cc)^a^	6.39	12.79	47.2 (18.5–92.6)
**3D-CRT-APBI**			
Lateral	0.57	1.48	4.2 (1.7–8.3)
Medial	0.51	1.34	3.8 (1.5–7.4)
**HDR MULTICATHETER**			
Lateral	1.44	4.28	10.6 (4.2–20.9)
Medial	1.58	4.67	11.7 (4.6–22.9)
**HDR BALLOON**			
Lateral	1.27	3.73	9.4 (3.7–18.4)
Medial	2.17	6.38	16.0 (6.3–31.5)

*^a^Large pendular breast treated wide tangents*.

*^b^Increased risk in major coronary events (myocardial infarction, coronary revascularization, and death from ischemic heart disease) is 7.4% (95% confidence interval 2.9–14.5%) per Gray (16)*.

*WBI, whole breast irradiation; 3D-CRT APBI, 3D-conformal radiation therapy accelerated partial breast irradiation*.

### Sensitivity analysis

We performed a sensitivity analysis. There was no significant difference in the value of the mean heart’s dose when the small and medium breast size phantoms were used. At the same time, its value doubled from 2.99 Gy to 6.39 Gy when the largest breast size phantom was used (Table 2). This was due to the posterior shift of the beam edge needed to fully cover the breast volume. The seroma location had little impact when using whole breast radiotherapy or 3D-CRT APBI. It increased the mean heart’s dose by 17% for a medially compared to laterally located seroma using multicatheter brachytherapy, and by 32% using balloon brachytherapy. This was essentially due to the closer proximity to the radioactive source. The most dramatic increase of mean heart’s dose was seen when we were testing set-up or organ motion errors for external beam radiotherapy. An anterior shift of the heart’s edge by 1.5 cm resulted in a 150% increase. For a large breast volume, the mean heart dose could reach 10.8 Gy, which corresponds to almost a twofolds increased risk of major coronary events. The set-up errors and organ motion effects were much less pronounced for brachytherapy techniques and this was the consequence of the smoother isodose gradient on the Gafchromic films anteriorly to the heart compared to external beam with heart shielding.

**Table 2:** **Set-up error and organ motion sensitivity analysis of the mean heart dose for the medium (800 cc) and large breast phantom (1200 cc) using a 1.5 cm anterior heart shift**.

Technique	Mean dose (Gy)	Relative to prescribed dose (%)	Increased risk of coronary events in % (95% CI)
**WBI**			
Medium (800 cc)	7.11	14.22	52.6 (20.6–100)
Large (1200 cc)^a^	10.79	21.59	79.8 (31.3–100)
**3D-CRT-APBI**			
Lateral	0.69	1.81	5.1 (2.0–10.0)
Medial	1.20	3.14	8.9 (3.5–17.4)
**HDR MULTICATHETER**			
Lateral	1.68	4.97	12.4 (4.9–24.4)
Medial	1.70	5.00	12.6 (4.9–24.7)
**HDR BALLOON**			
Lateral	1.34	3.96	9.9 (3.9–19.4)
Medial	2.44	7.19	18.1 (7.1–35.4)

*^a^Large pendulous breast treated wide tangents*.

*WBI, Whole breast irradiation; 3D-CRT APBI, 3D-conformal radiation therapy accelerated partial breast irradiation*.

## Discussion

This work reports the mean cardiac doses measured in an anthropometric phantom mimicking, a patient receiving breast radiotherapy with various techniques currently used for early stage breast cancer treatment. This study provides experimental data that could be considered more reliable compared to those calculated in commercial TPS. According to other publications, commercial TPS significantly underestimate the scatter dose outside the irradiation field (27, 35). Howell previously reported that the Eclipse’s analytic anisotropic algorithm gave a dose at the point 11.25 cm away from the treatment field border less by 55% than that of measured directly with thermoluminescent dosimeters (TLD) (35). To address this issue, our group used Monte Carlo simulation to estimate the dose delivered to the left anterior descending artery in an anthropometric phantom. Because secondary photons rarely crossed the volume of interest (VOI) the transport of a very large number of photons and multiple variance reduction strategies were necessary. Major simplifications have been made to the description of the phantom including large tally volumes (36).

Yet, since very low values are expected, the measurement of scattered dose remains challenging. The following quality assurance measures were undertaken to control the validity of our measurement. First, the doses at several points were checked using two independent methods, namely, the OSLDs and the Gafchromic films. Both are energy independent and the second one enables capturing a 3D spatial dose distribution stacking films. Second, we compared doses measured inside the high-dose treated volume and those calculated by the TPS. Those checks were considered satisfactory if they show differences in dose lower than 5%. Third, we repeated the experiments two times to ensure no major set-up error was made.

The most noteworthy finding of our study is that the mean heart dose was almost halved when using HDR APBI compared to WBI, even for the worst case scenario of a medially located left breast tumor. In this instance, the balloon brachytherapy technique does slightly worse, but still better than whole breast radiotherapy. The use of 3D-CRT APBI reduced the mean heart dose to one-third of what is received with use of HDR and to a sixth compared to WBI. This was essentially due to the limited extension of the posterior field border compared to WBI. It turns out the 3D-CRT APBI is the safest radiation technique and its use has the lowest risk of having major cardiovascular events. Those findings are consistent with previously reported ones. In a dose modeling study, Hiatt reported a sixfolds dose reduction when 3D-CRT was used instead of whole breast using IMRT (37). Also, Valach (25) reported a mean heart’s dose of 2.45 ± 0.94 Gy when balloon brachytherapy was used and the seroma was located in the inner quadrant of the left breast. Our measured value for a similar case was equal to 2.17 Gy.

One limitation of the present study is linked to the use of the Rando phantom that imposes estimating the 3D mean heart’s dose using only three Gafchromic films. Since the dose gradients are very smooth for the APBI technique, any impact would mainly concern the WBI technique where steep gradients are seen in the portion of the heart close to the beam edge. However, the films were placed perpendicular to the beam direction, such that a fine dose resolution was obtained in 2D. It is hence unlikely that any cold or hot spot may have been missed and that additional resolution would significantly change our findings. In addition, the main goal of the present study is not to provide exact value of mean heart’s dose since they could vary depending on many factors. The purpose of this study was mainly to compare different breast techniques, so using the same methodology enable a fair comparison among them. Another limitation of our work relates to the conversion of mean heart’s dose to major cardiovascular event risks for each radiation technique (18). The isodose fall-off in the heart is very different between WBI, HDR, and 3D-CRT APBI. It is much steeper for the first one and gradual for the other ones. This leads to very different DVH profiles and it is eventually unclear if comparing the mean instead of, for example, the median heart’s dose is the right approach. Identifying the critical structures involved in the radiation damage to the heart remains challenging. Coronary arteries including the left anterior descending artery or ventricles have been suggested (38). There is, however, no data correlating the doses received on those volumes to a prospectively evaluated clinical endpoint. We used the model proposed by Darby, as it remains the only one showing a statistically significant correlation between a risk of major cardiac events and a dosimetry parameter. But, we acknowledge that the risk we calculated for the various breast techniques maybe over or underestimated.

Using the predictive model proposed by Darby (18), a large variation in the values of the major coronary event risk is obtained. It ranges from a negligible 4–5% increase, in case of 3D-CRT and a medially or laterally located tumor, to a concerning 80% increase in case of a patient with a large breast having a systematic set-up error and/or motion exceeding 1.5 cm. This emphasizes the need of individual evaluation of risks accounting for potential intra-fraction errors and for patients with a large size breast with risk of set-up error measures to reduce the dose delivered to the heart must be taken. Those measures include gating the radiation delivery to the breathing cycle, using a prone position, 3D-CRT APBI technique, or proton therapy (39–41). Techniques like moderate deep inspiration breath hold have now been widely introduced into clinic. Although there is no long-term data to confirm its benefit in term of major cardiac event reduction, long-term experience shows that the mean heart’s dose is reduced by 40% (40).

It must be noted that although the finding of a better cardiac shielding using APBI is clearly appealing for a cancer population with excellent survival rates, the long-term outcomes of APBI remains unknown. If the early outcomes from large trials and multiple cohort studies appear promising (12, 13, 42, 43), a large population-based study shows contrariwise a marginal increased rate of mastectomy likely linked to local recurrence (44). It is eventually difficult to evaluate the final impact on the overall survival when balancing those opposite effects.

## Conflict of Interest Statement

The authors declare that the research was conducted in the absence of any commercial or financial relationships that could be construed as a potential conflict of interest.
